# Supplementary material for: Nurse-led secondary preventive follow-up after stroke/TIA and ACS for patients aged 80 years or older: A post-hoc analysis of the randomized controlled NAILED trial
Source: PLoS One. 2025 Nov 7;20(11):e0335930. doi: 10.1371/journal.pone.0335930 (PMC12594373; doi:10.1371/journal.pone.0335930)
Supplement: S1 File — (DOCX) [file pone.0335930.s001.docx]

**S1 Outcome definition. Definition of cardiovascular death.**

Cardiovascular death was defined as sudden cardiac death or death due to myocardial infarction, congestive heart failure, stroke, cardiovascular procedure, cardiovascular bleeding, or other cardiovascular conditions, such as peripheral artery disease or pulmonary embolism. Death in the first 30 days after myocardial infarction or stroke was considered death due to cardiovascular causes. Sudden cardiac death included unexpected death not caused by myocardial infarction, such as witnessed death 60 minutes after onset or worsening of cardiac symptoms, witnessed or unwitnessed death with identified arrhythmia, death after unsuccessful resuscitation from cardiac arrest, death after successful resuscitation from cardiac arrest when no non-cardiac etiology was evident, and death within 24 hours of last being observed stable with no signs of a non-cardiovascular cause. Cardiovascular death due to cardiovascular bleeding included non-stroke intracranial hemorrhage, non-traumatic and non-procedural vascular rupture, and cardiac tamponade.
