# Supplementary material for: Nurse-led secondary preventive follow-up after stroke/TIA and ACS for patients aged 80 years or older: A post-hoc analysis of the randomized controlled NAILED trial
Source: PLoS One. 2025 Nov 7;20(11):e0335930. doi: 10.1371/journal.pone.0335930 (PMC12594373; doi:10.1371/journal.pone.0335930)
Supplement: S1 Table — Categorical values are presented as N (%) and quantitative values as median (interquartile range). TIA, transient ischemic attack. (DOCX) [file pone.0335930.s002.docx]

|  | Completed active participation at the 3-year follow-up | |
| --- | --- | --- |
|  | **No** | **Yes** |
| N (%) | 84 (21.3) | 310 (78.7) |
| Women | 49 (58.3) | 143 (46.1) |
| Age, years | 85 (82.0-87.0) | 83 (81.0-87.0) |
| Qualifying event |  |  |
| Unstable angina | 1 (1.2) | 9 (2.9) |
| Myocardial infarction | 24 (28.6) | 157 (50.6) |
| Ischemic stroke | 33 (39.3) | 85 (27.4) |
| Intracerebral hemorrhage | 3 (3.6) | 8 (2.6) |
| TIA | 23 (27.4) | 51 (16.5) |
| Medical history | | |
| Atrial fibrillation | 31 (36.9) | 81 (26.1) |
| Diabetes | 13 (15.5) | 68 (21.9) |
| Hypertension | 65 (77.4) | 210 (67.7) |
| Endpoints during follow-up | | |
| Primary endpoint | 32 (38.1) | 104 (33.5) |
| Cardiovascular death | 14 (16.7) | 64 (20.6) |
| Myocardial infarction | 7 (8.3) | 34 (11.0) |
| Stroke | 20 (23.8) | 42 (13.5) |
| All-cause mortality | 38 (45.2) | 121 (39.0) |
| Ischemic stroke | 18 (21.4) | 36 (11.6) |
| Fracture | 27 (32.1) | 58 (18.7) |
| Serious bleeding | 6 (7.1) | 32 (10.3) |

**S2 Table. Comparison of patients who discontinued active participation before 3 years to those who participated for at least 3 years.**

Categorical values are presented as N (%) and quantitative values as median (interquartile range). TIA, transient ischemic attack.
