# Supplementary material for: Nurse-led secondary preventive follow-up after stroke/TIA and ACS for patients aged 80 years or older: A post-hoc analysis of the randomized controlled NAILED trial
Source: PLoS One. 2025 Nov 7;20(11):e0335930. doi: 10.1371/journal.pone.0335930 (PMC12594373; doi:10.1371/journal.pone.0335930)
Supplement: S2 File — (DOCX) [file pone.0335930.s003.docx]

**S3 Study protocol.**

**Research Plan**

**Secondary preventive follow-up after acute coronary syndrome (ACS) and stroke: nurse-led follow-up vs follow-up in primary healthcare - a randomized controlled study.**

**1. Purpose and Objectives**

According to statistics from the National Board for Health and Welfare 2007 (1) the most common mortality cause in Sweden, for both women (42%), and men (41%), is cardiovascular diseases. In the group cardiovascular disorders ischemic heart disease (including acute myocardial infarction, AMI) is responsible for 39% of the deaths in women and 50% in men. For stroke the respective percentages are 23% for women and 18% for men.

The incidence of AMI has decreased. Between the years 2001 and 2006 the standardised age incidence for men has decreased by 12% and for women by 7% (2). AMI is however still one of the most common causes for acute hospitalization in Sweden, comprising approximately 25 200 patients during 2007. According to RIKS-STROKE 2007 the number of hospitalized cases for stroke is almost as many; 24 130 patients (3).

Mortality due to myocardial infarction has decreased. Age standardised mortality due to myocardial infarction per 100 000 inhabitants has been reduced during the past 10-year period by an average of 4 % annually. AMI is still the single most common cause of mortality among women, 3 900 cases, and for men 4 900 cases. Corresponding number of deaths due to stroke (on all grounds) during 2007 for women was 4 600 and for men 3 300. There is also a decrease in incidence and mortality related to stroke (4).

The reasons for the decrease in mortality following myocardial infarction is considered partly to be due to the lower incidence and partly to the ever improving efficiency in the treatment of myocardial infarction (5). The fact that the incidence of myocardial infarction is seen to be decreasing can be accredited to a more favourable risk factor profile in the population (4). The serious risk factors such as smoking, high blood pressure and high cholesterol, have improved during latter years. This is probably also the reason for the decreased incidence of stroke.

Secondary preventive measures contribute to improved risk factor profiles and the decreased incidence of myocardial infarction and stroke. Secondary prevention can also include measures to induce a favourable influence on smoking cessation, diet, physical activity, stress and psychosocial factors as well as encouraging the use of prescribed medication intended to reduce the risk of relapse. Effective secondary prevention can lead to a tangible reduction of mortality and relapse. Smoking cessation post myocardial infarction can e.g., after a year, lead to a relative risk reduction of 50 percent in mortality and relapse (6).

Medication such as acetylsalicylic acid, statins and beta blockers following a myocardial infarction have been seen to lead to a relative risk reduction for both mortality and relapse to the extent of 20-30% (7). A similar risk reduction for relapse and mortality has been noted when antiplatelet, antihypertensive and lipid-lowering medication is prescribed following a stroke (8). Secondary preventive intervention is imperative for reducing mortality following a myocardial infarction, much more important than e.g. invasive treatment (9).

May we then presume that current practical application of secondary preventive treatments following a myocardial infarction or stroke is effective? The answer is unfortunately negative from a national and international perspective. The EUROASPIRE overviews maintain that the risk factor impact in the aftermath of a myocardial infarction is highly unsatisfactory (10). During the past 12 years, despite an increase in the use of prescribed secondary preventive medication, the percentage of patients achieving the set objectives regarding blood pressure, the number of diabetics with a satisfactory glycemic control and the number of patients that have stopped smoking have not improved. Lipid levels have improved but at the same time a tangible increase of obesity and diabetes has been noted. There is a substantial discrepancy between the standard promoted in guidelines and the actual result achieved in clinical practice.

The quality of secondary preventive care following a myocardial infarction in Sweden is evaluated in the SEPHIA register (5). At a follow-up after one year the percentage of patients who had achieved the set objective regarding smoking cessation was 59%, systolic blood pressure 78%, low density lipoprotein cholesterol barely 70% and physical activity 43%. A summarized quality measure comprising 5 objectives was achieved by 19% of the patients after one year.

Regarding secondary prevention post stroke knowledge as to how the various treatments are applied is highly unsatisfactory. There is no systematic follow-up at either Swedish or European level, but a few reports regarding trials with more structured follow-up have been published (11). A small project, including 328 patients from RIKS-STROKE revealed that only one third of the patients had achieved the set blood pressure objective after a 3 month follow-up. Other cardiovascular risk factors had been inadequately recorded and could not be evaluated (12).

Accordingly, there is enormous potential for improved secondary preventive care, especially since available data overestimates the results of treatment. This is partly due to the fact that a substantial percentage of patients in the intended target group are not included in the reports and these patients usually attain poorer results. Age limits are applied, EUROASPIRE sets the age limit at 70 years of age and SEPHIA has an age limit of 75 years. This means that almost 50% of the myocardial infarction patients are excluded from the evaluations and it is often more difficult for older patients to achieve the risk factor objectives (13).

Currently large drug trials are being implemented, each and every requiring huge investments with an expected absolute risk reduction regarding morbidity and mortality, at less than one percentage annually (14, 15). The potential profit of a more effective secondary prevention is much higher at a fraction of the cost.

Therefore, we plan to implement a randomised study regarding secondary prevention following a myocardial infarct or stroke, where the follow-up will be based on procedures led by specially trained nurses (intervention group) and compared with the current follow-up routines in Sweden, carried out in the primary healthcare (control group). Unique elements in the study are: 1) All patients with acute coronary syndrome (ACS) or stroke will be included, irrespective of age. Inclusion will be ongoing during the entire year, i.e. all patients with an ACS or stroke during the inclusion phase will be asked to participate. 2) Population based. All patients in the county of Jämtland will be included, i.e. those who are admitted to Östersund Hospital. 3) The nurses working with the intervention group must have adequate training and delegation to titrate statin doses. The contact with patients will be telephone-based. Focus will be on maximizing advantages and minimizing cost. 4) A long follow-up period. The majority of studies have a follow-up period of one or two years. For patients enrolled in the beginning of the inclusion period we plan for a follow-up of 5 years. The outcome will reflect a period of time relevant from the perspective of both patients and the healthcare system.

**2. Hypotheses**

Nurse-led, telephone-based secondary preventive follow-up after ACS or stroke, focusing on set objectives for lipids and blood pressure, smoking cessation and counselling/support regarding physical activity and diet, increase objective achievement, reduce morbidity/mortality is more cost effective than traditional follow-up conducted in the primary healthcare.

**3. Research questions**

Compared with primary healthcare based secondary preventive follow-up after ACS and stroke, do nurse-based secondary preventive follow-up result in:

1. A more satisfactory prognosis regarding cardiovascular morbidity and overall mortality?
2. Improved compliance regarding the use of prescribed medication?
3. Better achievement of set objectives regarding cardiovascular risk factors?
4. A better cost efficiency?

Have the socioeconomic and stress related factors influenced:

1. The prognosis regarding cardiovascular morbidity and overall mortality?
2. Compliance regarding the use of prescribed medication?
3. Target achievement regarding cardiovascular factors?

**4. Methods including substudies**

**4.1 Definitions**

4.1.1 Definition ACS

1. Acute myocardial infarct type 1 according to the consensus document Circulation 2007/2012 (8,9).
2. Unstable angina, typical ischemic symptoms in combination with dynamic ECG deviations (ST and/or T deviations) characteristic for ischemia.

4.1.2 Definition stroke

According to WHO: rapid development of clinical signs of focal (or global) disturbance of cerebral function with a duration surpassing 24 hours (if the process is not terminated due to mortality or surgery) and has no other explanation than vascular symptoms. A corresponding occurrence with duration less than 24 hours is defined as a transient ischemic attack (TIA).

4.1.3 Definition of intervention

Telephone-based monitoring and bloods after 6-10 weeks, 1, 2, 3, 4 and 5 years after an ACS respectively stroke/TIA, supervised by a nurse, who after specialized training can be delegated to titrate statin medication and modify antihypertensive treatment as instructed. Questions arising which are not covered by the standardised procedure are referred to a physician, who then contacts the patient if this is considered necessary. Modification of medication strives to achieve the desired objectives as soon as possible. Objective for blood pressure: seated systolic <130 – 140, seated diastolic <80 – 90 according to the revised guidelines stated by the European Society of Hypertension November 2009 (10). Objective for lipids: cholesterol <4.5, LDL <2,5. Counselling/support regarding smoking cessation and when necessary further contact via a smoking cessation clinic. Counselling/support regarding physical activity and diet. Bloods and diagnostic measurements are usually carried out at the nearest health center alternatively district nurse clinic according to common clinical routines.

4.1.4 Definition of control

Routine check-ups will be performed via the primary health care, to which the patient is referred when no further measures are called for at the coronary respectively stroke unit. In most cases this implies referral directly on discharge from hospital or after one or two initial follow-up appointments at the unit. This is consistent with the most common current routine in Sweden and the given times for reporting to on-going registration to the national quality resister SEPHIA. Risk factor objectives are assessed at each respective healthcare center.

**4.2 Inclusion criteria**

1. Acute myocardial infarct
2. Unstable angina according to the definition 4.1.1
3. Acute stroke or TIA according to the definition 4.1.2

**4.3 Exclusion criteria**

1. Mentally incapable of participation, due to dementia, mental illness, terminal illness.
2. Unable to communicate by telephone due to e. g. hearing disabilities or aphasia.
3. Patients with complicated illnesses requiring continued monitoring via specialists.
4. Patients with subarachnoid or subdural haemorrhage.

**4.4 Intervention**

According to definition 4.1.3

Randomization to the intervention or control group, respectively.

**4.5 Blood tests and examinations**

4.5.1 At inclusion routine samples during the initial hospital admission.

Clinical Chemistry and Laboratory Medicine (Clin Chem Lab Med):

Routine hematology, Electrolyte status, high-sensitivity C-reactive protein (hs-CRP), lipids, fP-glucose, Hemogobin A1c (HbA1c)

Physical measurement diagnostics:

Blood pressure (seated and standing), electrocardiography (ECG)

Others:

Weight, height, abdominal circumference

Socioeconomic factors ([www.scb.se/sei](http://www.scb.se/sei))

Surveys pertaining to stress related factors according to Center for environmentally related illnesses and stress (CEOS).

Current medication.

4.5.2 After 6 – 10 weeks

Clin Chem Lab Med:

Lipids, HbA1c – if previously atypical

Physical measurement diagnostics:

Blood pressure (seated and standing)

Others:

Weight

Current medication.

Number of health care contacts, hospital care, diagnosis, number of days in hospital, number of days on sick leave

Medication titration

Physical activity and smoking

4.5.3 – 4.5.7 After 1, 2, 3, 4 and 5 years

Clin Chem Lab Med:

Lipids, HbA1c – if previously atypical

Physical measurement diagnostics:

Blood pressure (seated and standing), ECG

Others:

Weight, abdominal circumference

Current medication and dosage

Hospital care, diagnosis, number of days in hospital

Medication titration

Physical activity and smoking

**4.6 Outcome variables**

4.6.1 Prognosis study

Primary: Mortality, relapse of myocardial infarct/stroke.

Secondary: Separate assessment of the individual components of the primary endpoint; hospital care: admittance and number of days in hospital.

4.6.2 Compliance study

Primary: Percentage of patients taking prescribed medication at the various times of follow-up (anti-platelet, anticoagulant, lipid-lowering and antihypertensive medication). Intervention group versus the control group.

Analysis of non-compliance according to Osterberg Blaschke (17).

4.6.3 Study of risk factor objectives

Primary: Percentage of patients that achieved the set objectives at each respective time regarding lipids and blood pressure.

Secondary: Absolute values at each respective follow-up time regarding lipids and blood pressure. Intervention group versus the control group.

An analysis concerning the extent of the healthcare inputs necessary to achieve the set objectives: number of appointments, telephone contacts and titration occasions should be registered.

An analysis regarding the need of drugs to improve blood pressure and lipids: number of prescriptions, dosage, the necessity for more potent statins, all in relation to age and sex.

4.6.4 Cost-effective study

Primary: The cost per quality adjusted life year (QUALY).

Secondary: the actual cost per patient in each respective group.

4.6.5 Stress-related factors and prognosis

Primary: Morbidity and mortality in relation to variables in the surveys.

Secondary: Compliance and achieved objectives in relation to variables in the surveys.

4.6.6 Socioeconomics and prognosis

Primary: Morbidity and mortality in relation to the socioeconomics classification according to SEI ([www.scb.se/sei](http://www.scb.se/sei)).

Secondary: Compliance and achieved objectives in relation to SEI classification.

**4.7 Statistics**

4.7.1 Size of the study group

4.7.1.1 Prognosis study primary end points

In a recently implemented study (18), with similar inclusion criteria regarding myocardial infarct (number of included subjects was 534) we found that the incidence of death, AMI and stroke at 2 years follow-up was approximately 50%. The expected frequency of these events will be around 60% after an average follow-up time of 3 – 4 years. A study comprising 300 patients in each respective group is then able to detect a risk reduction of 20 percentage point with 80% power and a significance level of 0.05.

A planned analysis, including both ACS and stroke patients, will consists of study groups comprising approximately 600 patients. A risk reduction of approximately 7- 8 percentage point can then be detected with 80% power and a significance level of 0.05.

4.7.2 Other analyses

4.7.2.1 Primary end points

Parallel groups, estimation of relative risk with a 95% confidence interval and hazard ratio through Cox regression. Kaplan Meyer analysis with the log-rank test. Adjustment for baseline variables, if necessary, by a Cox regression model.

4.7.2.2 Secondary end points

Please see 4.8.2.1

4.7.2.3 Compliance analysis

At the various time points of follow-up, the percentage of patients in the intervention and control groups that follows respective ordinations; Chi-2 test. The Mann-Whitney test is used to compare the percentage of compliance regarding all cardiovascular medication for each patient in the intervention respectively control groups.

4.7.2.4 Analysis of risk factor objectives

Primary, the percentage of patients who have achieved the set objectives: Chi2 test.

Secondary, absolute values: T-test, independent groups.

4.7.2.5 Stress related factors

Dichotomized variables. Primary analysis as in 4.8.2.3

Secondary analysis regarding compliance and target values as in 4.8.2.3 respectively 4.8.2.4

4.7.2.6 Socioeconomics analysis

Deployment of SEI-classes. Kaplan Meyer analysis regarding primary and secondary outcomes. Multivariate Cox regression analysis for identification of variables (including SEI-classes) that predict primary outcome. Chi2 test regarding percentage of compliance and percentage of achieved objectives.

**5. Preliminary results**

In the KAPRIS study a nurse-based secondary preventive treatment model has been on trial at the Cardiac Unit, Östersund Hospital, Sweden. Preliminary results were presented at the Medical Association National Conference 2008 (19) and the results after a long term follow-up will be presented at the above mentioned National Conference for the year 2009. Data show that compliance, risk factor levels and morbidity can be substantially improved when using this model. Despite being merely observation data without a strict inclusion routine it presents a convincing basis for the planned randomised study, which will adhere to a strict protocol.

**6. Significance**

**6.1 Prognosis study**

Provides an opportunity to measure the outcome of serious incidents (mortality, stroke, myocardial infarct) directly between a follow-up routine based on specialist nurse supervision respectively current prevalent routines in primary healthcare. We know that secondary preventive measures are currently not optimal. The reasons for this are not entirely clear but factors such as staffing problems, numerous care givers being involved in the chain of decision-making, financial problems, overloading at all levels of the health care system, the need to prioritize, problems with logistics etc all play their part in this. A favourable result of an organizationally straightforward nurse based secondary prevention with a lower outcome of morbidity and mortality would motivate implementation of this model as well as allowing for a permanent status. Significant positive effects regarding both health (morbidity and mortality) and economics would be foreseeable.

**6.2 Compliance**

Knowledge regarding adherence to prescribed medication and the reasons why prescriptions are not followed is highly inadequate. Available data show that approximately 50% of the patients have problems in following a given prescription within a period of 5 years. Most of the resulting problems could probably be solved and the effect of the treatment be retained. The study will show if access to and monitoring by a specialist nurse can enhance compliance and the effect of the treatment. The population aspect, i.e. the inclusion of all patients within a specific catchment area gives the study a unique strength regarding generalizability.

**6.3 Risk factor values**

Guidelines recommend target values regarding lipids and blood pressure following a myocardial infarct respectively stroke. The objectives are set according to results from large intervention studies. The target values for lipids have become more challenging and require significant measures to achieve. We have currently no reliable data that explains the prerequisites regarding patient contact, dosage titration, and adjustment of prescriptions etc. needed to achieve the current objectives. Knowledge pertaining to objectives and the necessary measures to achieve them are more or less non-existent regarding the group of older patients (>80 years of age) who dominate entirely regarding morbidity and mortality. The study will provide a solid bank of knowledge in this field.

**6.4 Cost effective study**

Provides a way to measure the financial effects of the intervention. It is measured partly by QUALYs and partly by directly measuring the actual cost alternatively saving per patient. Myocardial infarct and stroke are extremely costly for the healthcare system; consequently a reduction of these incidences has the potential to save a substantial amount of money, which can be calculated accurately in the study.

**6.5 Stress related factors and prognosis**

Population based, prospective data showing stress related variable’s significance for morbidity and mortality following a myocardial infarct or stroke is not currently available.

**6.6 Socioeconomics and prognosis**

Knowledge about socioeconomic factors’ significance for morbidity, mortality, compliance and risk factor levels is highly inadequate, which has recently been commented by the National Board for Health and Welfare. A socioeconomic survey in the study provides grounds to improve this lack of knowledge.

**7. Timetable**

The study will be implemented during the period January 4^th^ 2010 through December 31^st^ 2014.

**8. Referenser**

1. Socialstyrelsen. Dödsorsaker 2007. Socialstyrelsen 2009.

2. Socialstyrelsen. Hjärtinfarkter 1987-2006. Socialstyrelsen 2008.

3. Årsrapport Riks-Stroke 2007. Riks-Stroke 2008.

4. Socialstyrelsen. Folkhälsorapport 2009. Socialstyrelsen 2009.

5. Stenestrand U, Wallentin L, Lindahl B, Tyden P, Hambraeus K, James S, et al. Årsrapport 2007 - RIKS-HIA, SEPHIA och SCAAR. UCR, Uppsala.; 2008.

6. Wilhelmsson C, Vedin JA, Elmfeldt D, Tibblin G, Wilhelmsen L. Smoking and myocardial infarction. Lancet 1975;2:1157-60.

7. Rockson SG, deGoma EM, Fonarow GC. Reinforcing a continuum of care: in-hospital initiation of long-term secondary prevention following acute coronary syndromes. Cardiovasc Drugs Ther 2007;21(5):375-88.

8. Talelli P, Greenwood RJ. Recurrent stroke: where do we stand with the secondary prevention of noncardioembolic ischaemic strokes? Ther Adv Cardiovasc Dis 2008;2(5):387-405.

9. Bjorck L, Rosengren A, Bennett K, Lappas G, Capewell S. Modelling the decreasing coronary heart disease mortality in Sweden between 1986 and 2002. Eur Heart J 2009;30(9):1046-56.

10. Kotseva K, Wood D, De Backer G, De Bacquer D, Pyorala K, Keil U. Cardiovascular prevention guidelines in daily practice: a comparison of EUROASPIRE I, II, and III surveys in eight European countries. Lancet 2009;373(9667):929-40.

11. Bushnell C, Zimmer L, Schwamm L, Goldstein LB, Clapp-Channing N, Harding T, et al. The Adherence eValuation After Ischemic Stroke Longitudinal (AVAIL) registry: design, rationale, and baseline patient characteristics. Am Heart J 2009;157(3):428-435 e2.

12. Collen A-C, Lagerlöf A, Nieburg I, Carlberg B. Blodtryck efter stroke - hur många når mål? Svenska Läkaresällskapets Riksstämma 2008;Internmedicin:6P.

13. Hanna IR, Wenger NK. Secondary prevention of coronary heart disease in elderly patients. Am Fam Physician 2005;71(12):2289-96.

14. Barter PJ, Caulfield M, Eriksson M, Grundy SM, Kastelein JJ, Komajda M, et al. Effects of torcetrapib in patients at high risk for coronary events. N Engl J Med 2007;357(21):2109-22.

15. Bhatt DL, Fox KA, Hacke W, Berger PB, Black HR, Boden WE, et al. Clopidogrel and aspirin versus aspirin alone for the prevention of atherothrombotic events. N Engl J Med 2006;354(16):1706-17.

16. Thygesen K, Alpert JS, White HD, Jaffe AS, Apple FS, Galvani M, et al. Universal definition of myocardial infarction. Circulation 2007;116(22):2634-53.

17. Osterberg L, Blaschke T. Adherence to medication. N Engl J Med 2005;353(5):487-97.

18. Modica A, Karlsson F, Mooe T. The impact of platelet function or C-reactive protein, on cardiovascular events after an acute myocardial infarction. Thromb J 2009;7:12.

19. Ohlsson-Önerud Å, Mooe T, Modica A, Friberg B, Persson K. KAPRIS - ett Kardiovaskulärt Prevention och Risk Intervenerande System. Läkaresällskapets Riksstämma 2008;Internmedicin:17P.

# Forskningsplan

# Sekundärpreventiv uppföljning efter akut koronart syndrom (AKS) eller stroke via specialistsjuksköterska eller primärvård - en randomiserad studie.

**1. Bakgrund och relation till tidigare forskning**

Hjärt- och kärlsjukdomar är i Sverige den vanligaste dödsorsaken för såväl kvinnor, 42%, som män, 41%, enligt Socialstyrelsens statistik för 2007 (1). Inom gruppen hjärt- och kärlsjukdomar står ischemisk hjärtsjukdom (inkluderar akut hjärtinfarkt) för 39% av dödsfallen hos kvinnor och 50% av dödsfallen hos män. För stroke är respektive andel 23% för kvinnor och 18% för män.

Incidensen av akut hjärtinfarkt har sedan länge minskat. Mellan år 2001 och 2006 har den åldersstandardiserade incidensen för män minskat med 12% samt för kvinnor med 7% (2). Akut hjärtinfarkt är dock fortfarande den vanligaste orsaken till akut sjukhusvård i Sverige, ca 25200 patienter under år 2007. Antalet vårdtillfällen för stroke är nästan lika många, enligt RIKS-STROKE 2007 24 130 (3).

Mortaliteten i hjärtinfarkt minskar. Den åldersstandardiserade dödligheten i akut hjärtinfarkt per 100000 invånare har under den senaste 10-årsperioden sjunkit med i genomsnitt 4 procent årligen. Akut hjärtinfarkt är fortfarande den enskilt vanligaste dödsorsaken såväl hos kvinnor, 3900 fall, som hos män, 4900 fall. Motsvarande antal avlidna p.g.a stroke (alla orsaker) var 2007 för kvinnor 4600 och för män 3300. Även när det gäller stroke minskar incidens och mortalitet (4).

Orsaker till den minskande mortaliteten i hjärtinfarkt anses dels vara den lägre incidiensen dels en allt effektivare behandling i samband med hjärtinfarkt (5). Att incidensen av hjärtinfarkt minskar kan tillskrivas en gynnsammare riskfaktorprofil i befolkningen (4). De tunga riskfaktorerna rökning, högt blodtryck och höga kolesterolvärden har förbättrats under senare år. Detta ligger sannolikt även bakom den minskade incidensen av stroke.

Till en bättre riskfaktorprofil och den minskade incidensen av hjärtinfarkt och stroke bidrar det sekundärpreventiva arbetet. Sekundärprevention kan omfatta insatser för att gynnsamt påverka rökning, kost, motion, stress och psykosociala faktorer samt användning av läkemedel som minskar risken för återinsjuknande. Med ett effektivt sekundärpreventivt arbete kan en påtaglig reduktion av död och återinsjuknande åstadkommas. Rökstopp efter hjärtinfarkt kan t.ex. efter ett år ge en 50%-ig relativ riskreduktion av död och reinfarkt (6).

Läkemedel som ASA, statiner och betablockerare efter hjärtinfarkt har visats ge en relativ riskreduktion för död och reinfarkt i området 20-30% (7). Liknande riskminskning för återinsjuknande och död ses vid behandling med tromboshämmande, blodtryckssänkande och blodfettssänkande medel efter stroke (8). Sekundärprevention har nyligen visats vara den behandlingsmetod som varit viktigast för att minska mortaliteten efter hjärtinfarkt, betydligt viktigare än t.ex. invasiva behandlingar (9).

Är då dagens praktiska tillämpning av sekundärpreventiv behandling efter hjärtinfarkt respektive stroke effektiv? Svaret blir tyvärr nej såväl i ett nationellt som i ett internationellt perspektiv. I EUROASPIRE översikterna konstateras att riskfaktorbilden efter hjärtinfarkt i ett antal europeiska länder är en besvikelse (10). Under de senaste 12 åren har visserligen sekundärpreventiv läkemedelsanvändning ökat men andelen patienter som uppnår målvärden avseende blodtryck, andelen diabetiker med god glykemisk kontroll och andelen som slutar röka har inte förbättrats. En ljuspunkt är att lipidnivåerna har förbättrats men samtidigt ses en klar ökning av fetma och diabetes. Det finns en påtaglig diskrepans mellan den standard som förespråkas i guidelines och det resultat som uppnås i praktiskt sjukvård.

I Sverige värderas den sekundärpreventiva kvaliteten efter hjärtinfarkt i SEPHIA registret (5). Vid uppföljning efter ett år var andelen patienter som nådde målvärden avseende rökstopp 59%, systoliskt blodtryck 78%, LDL-kolesterol knappt 70% och fysisk träning 43%. Ett sammanfattande kvalitetsmått innehållande 5 målvärden uppnåddes helt hos 19% av patienterna efter ett år.

När det gäller sekundärprevention efter stroke är kunskapen om hur olika behandlingar tillämpas mycket bristfällig. Det finns ingen systematisk uppföljning varken på nationell nivå i Sverige eller på Europanivå men det har nu publicerats enstaka exempel på försök till mer strukturerad uppföljning (11). I ett mindre projekt inkluderande 328 patienter från RIKS-STROKE konstaterades att endast 1/3 av patienterna hade nått målblodtryck efter 3 månaders uppföljning samt att övriga kardiovaskulära riskfaktorer var undermåligt kontrollerade och inte kunde värderas (12).

Det finns således en stor potential för förbättrad sekundärprevention i synnerhet som befintliga data överskattar behandlingsresultatet. Det beror dels på att en stor andel patienter i avsedd målgrupp inte ingår i redovisningarna och dessa patienter uppnår som regel ett sämre resultat. Dels används åldersgränser, i EUROASPIRE 70 år och i SEPHIA 75 år. Detta innebär att närmare 50% av hjärtinfarktpatienterna står utanför utvärderingarna och hos de äldre kan det vara svårare att nå aktuella målvärden (13).

Det genomförs i dag stora läkemedelsprövningar som var och en kräver investeringar på nivån miljarder kronor med en förväntad absolut vinst vad gäller kardiell morbiditet och mortalitet på mindre än en procentenhet per år (14, 15). Den potentiella vinsten vid effektivare sekundärprevention ligger flerfaldigt högre till en bråkdel av kostnaden.

Vi planerar därför att genomföra en randomiserad studie avseende sekundärprevention efter hjärtinfarkt och stroke där uppföljning baserad på insats av särskilt utbildad sjuksköterska (interventionsgrupp) jämförs med den i Sverige vanliga uppföljningen inom primärvården (kontrollgrupp).

Unika inslag i studien är: 1) Samtliga patienter med akut koronart syndrom (AKS) eller stroke inkluderas, oberoende av ålder. Inklusion under årets samtliga dagar d.v.s. samtliga patienter som insjuknar under inklusionsperioden tillfrågas om deltagande. 2) Populationsbaserad. Inklusion av samtliga patienter som insjuknar inom Jämtlands län, d.v.s. vårdas på Östersunds sjukhus. 3) De sjuksköterskor som följer upp interventionsgruppen har utbildning och delegering för att titrera doser på statiner. Uppföljning via telefonkontakt. Innebär fokusering på att maximera nytta och minimera kostnad. 4) Lång uppföljningstid. De flesta studier har en uppföljningstid på enstaka år. Vi planerar för en 5-årig uppföljning av de först inkluderade patienterna. Resultatet kommer då att spegla en tidsrymd som är relevant ur patientens perspektiv men även ur ett sjukvårdsperspektiv.

**2. Hypoteser**

Sjuksköterskebaserad sekundärpreventiv uppföljning via telefon efter AKS och stroke fokuserad på målvärden för lipider och blodtryck samt rökstopp och rådgivning avseende motion och kost ökar måluppfyllelse och minskar morbiditet/mortalitet och är mer kostnadseffektivt än traditionell uppföljning i primärvården.

**3. Frågeställningar**

Medför sjuksköterskebaserad jämfört med primärvårdsbaserad sekundärpreventiv uppföljning efter AKS och stroke:

1. Bättre prognos avseende kardiovaskulär morbiditet och total mortalitet?
2. Bättre kompliance avseende läkemedelsanvändning?
3. Bättre måluppfyllelse avseende kardiovaskulära riskfaktorer?
4. Bättre kostnadseffektivitet?

Har socioekonomiska och stressrelaterade faktorer inverkan på:

1. Prognos avseende kardiovaskulär morbiditet och total mortalitet?
2. Kompliance avseende läkemedelsanvändning?
3. Måluppfyllelse avseende kardiovaskulära riskfaktorer?

**4. Metod inkluderande delstudier**

**4.1 Definitioner**

4.1.1 Definition akut koronart syndrom:

1. Akut hjärtinfarkt typ 1 enligt konsensusdokument Circulation 2007 (16).
2. Instabilt angina. Typiska ischemiska symtom i kombination med dynamiska EKG förändringar (ST- och/eller T-förändringar) talande för ischemi.

4.1.2 Definition stroke

Enligt WHO: snabbt utvecklade kliniska tecken på fokal (eller global) störning av cerebral funktion med duration mer än 24 timmar (om förloppet ej avbryts av död eller kirurgisk åtgärd) utan annan förklaring än vaskulär bakgrund. En motsvarande händelse med duration mindre än 24 timmar definieras som TIA (transitorisk ischemisk attack).

4.1.3 Definition intervention

Uppföljning per telefon 6-10 veckor, 12 månader, 2, 3, 4 och 5 år efter AKS respektive stroke av sjuksköterska som, efter utbildning, har delegation för att titrera statinbehandling. Vid behov tas extra telefonkontakt i samband med läkemedelstitreringar. Frågeställningar som inte ryms inom den standardiserade handläggningen diskuteras med läkare som även kontaktar patienten om så är medicinskt motiverat. Justering av läkemedel syftar till att så snart möjligt uppnå aktuella målvärden. Rådgivning avseende rökstopp och vid behov fortsatt kontakt via rökavvänjningsmottagning. Rådgivning avseende motion och kost. Aktuella provtagningar och mätningar görs som regel via närmaste vårdcentral alternativt distriktssköterska motsvarande vanlig klinisk rutin.

4.1.4 Definition kontroll

Uppföljning via primärvården dit patienten remitteras när ingen ytterligare åtgärd är aktuell vid hjärtenheten respektive strokeenheten. I de flesta fall innebär det remittering direkt vid utskrivning eller efter ett initialt uppföljande återbesök. Provtagning enligt gällande rutin samt vid de tidpunkter som gäller för rapportering till pågående nationellt kvalitetsregister, SEPHIA. Resultaten bedöms på vederbörande vårdcentral.

**4.2 Inklusionskriterier**

1. Akut hjärtinfarkt
2. Instabil angina enligt definition 4.1.1 ovan.
3. Akut stroke eller TIA enligt definition 4.1.2 ovan.

**4.3 Exklusionskriterier**

1. Mental oförmåga att delta, dvs. demens, psykisk sjukdom, terminal sjukdom och motsv.
2. Oförmåga att kommunicera per telefon, t.ex. kraftigt nedsatt hörsel
3. Patienter med komplicerad sjukdomsbild som kräver fortsatt uppföljning på specialistmottagning
4. Patienter med subarachnoidalblödning eller subduralblödning

**4.4 Intervention**

Enligt definition 4.1.3

Randomisering till interventionsgrupp respektive kontrollgrupp.

**4.5 Provtagning och undersökningar**

4.5.1 Vid inklusion rutinprover under vårdtillfället:

Kem.lab.:

Blodstatus, elektrolytstatus, hsCRP, lipidstatus, fP-glukos, HbA1c

Fys.lab.:

Blodtryck sittande och stående, EKG

Övrigt:

Vikt, längd, bukomfång

Socioekonomiska faktorer ([www.scb.se/sei](http://www.scb.se/sei))

Enkät avseende stressrelaterade faktorer enligt CEOS (Centrum för miljörelaterad ohälsa och stress)

Aktuella läkemedel och doser

4.5.2 Efter 6-10 veckor

Kem.lab:

Lipidstatus, HbA1c om tidigare avvikande

Fys.lab:

Blodtryck sittande och stående

Övrigt:

Vikt

Aktuella läkemedel och doser

Vårdkontakter, sjukhusvård, diagnos, dagar, sjukskrivningsdagar

Läkemedelstitreringar

Fysiskt aktivitet och rökning

4.5.3 - 4.5.7 Efter 1, 2, 3, 4 och 5 år

Kem.lab:

Lipidstatus, HbA1c om tidigare avvikande

Fys.lab:

Blodtryck sittande och stående, EKG

Övrigt:

Vikt, bukomfång

Aktuella läkemedel och doser

Vårdkontakter, sjukhusvård, diagnos, dagar

Läkemedelstitreringar

Fysiskt aktivitet och rökning

**4.6 Effektvariabler**

4.6.1 Prognosstudie

Primärt: Död, reinfarkt, stroke

Sekundärt: enskilda primära mätpunkter; sjukhusvård: inläggningar och vårddagar

4.6.2 Compliancestudie

Primärt: Procentandel som tar respektive ordinerat läkemedel vid de olika tidpunkterna (trombocythämmare, antikoagulantia, lipidsänkare, blodtrycksläkemedel). Analys intervention vs kontrollgrupp.

Fördjupad analys med sammanställning av orsaker till non-compliance enligt Osterberg Blaschke (17).

4.6.3 Målvärdesstudie

Primärt: Andel med uppnådda målvärden vid respektive tidpunkt avsseende lipider och blodtryck.

Sekundärt: Absoluta värden vid respektive tidpunkt avsseende lipider och blodtryck.

Analys intervention vs kontrollgrupp.

Fördjupad analys av omfattning av vårdinsats för att nå målvärden: antal besök, telefonkontakter och antal titreringstillfällen registreras.

Fördjupad analys avseende läkemedelsbehov för kontroll av blodtryck och lipider: antal preparat, doser, behov av potentare statin, relation till ålder och kön.

4.6.4 Kostnads-effektivitetsstudie

Primärt: Kostnad per QUALY (quality adjusted life year).

Sekundärt: Faktisk kostnad per patient i respektive grupp.

4.6.5 Stressrelaterade faktorer och prognos

Primärt: Morbiditet och mortalitet i relation till variabler i stressenkät.

Sekundärt: Kompliance och uppnådda målvärden i relation till variabler i stressenkät.

4.6.6 Socioekonomi och prognos

Primärt: Morbiditet och mortalitet i relation till socioekonomiska indelning enl. SEI ([www.scb.se/sei](http://www.scb.se/sei)).

Sekundärt: Kompliance och uppnådda målvärden i relation till socioekonomisk indelning.

**4.7 Statistik**

4.7.1 Storlek på studiegrupp

4.7.1.1 Prognosstudie primär effektvariabel

I en av oss nyligen genomförd studie (18) med likartade inklusionskriterier avseende hjärtinfarkt (antal inkluderade hjärtinfarktpatienter var 534) var incidensen av död, reinfarkt och stroke vid 2 års uppföljning ca 50%. En konservativ uppskattning ger en frekvens av dessa händelser på ca 60% efter en genomsnittlig uppföljningstid på 3-4 år. En studiestorlek på 300 patienter i respektive grupp kan då detektera en riskreduktion på 20 procentenheter med 80% power och signifikansnivå 0.05.

Planerad analys med inklusion av såväl strokepatienter som patienter med AKS ger studiegrupper omfattande ca 600 patienter. En riskreduktion på ca 7-8 % enheter kan då detekteras med 80% power och signifikansnivå 0.05.

4.7.2 Övriga analyser

4.7.2.1 Primär effektvariabel

Parallella grupper, beräkning av relativ risk med 95% konfidensintervall samt hazard ratio via Cox regression. Kaplan Meyer analys med log-rank test. Justering av eventuella skillnader i baslinjevariabler med Cox regressions modell.

4.7.2.2 Sekundära effektvariabler

Som 4.8.2.1

4.7.2.3 Kompliance analys

Jämförelse mellan interventions och kontrollgrupp vid de olika kontrolltidpunkterna med Chi-2 test, andel i respektive grupp som följer respektive ordination. Procentandel kompliance avseende samtliga hjärt-kärl läkemedel för varje patient jämförs mellan interventions- och kontrollgrupp med Mann-Whitneys test.

4.7.2.4 Målvärdesanalys

Primärt, andel med uppnådda målvärden. Chi2 test.

Sekundärt, absoluta värden. T-test, oberoende grupper.

4.7.2.5 Stressrelaterade faktorer

Dikotomiserade variabler. Primär analys som 4.8.2.1

Sekundär analys angående kompliance och målvärden som 4.8.2.3 respektive 4.8.2.4

4.7.2.6 Socioekonomisk analys

Gruppering av sei-klasser. Kaplan Meyer analys avseende primärt och sekundära effektmått. Multivariat Cox regressionsanalys för identifiering av variabler (inkluderande sei-klasser) som predicerar primärt effektmått. Chi2 test avseende andel kompliance samt andel med uppnådda målvärden.

**5. Preliminära resultat**

I den s.k. KAPRIS studien har en sjuksköterskebaserad sekundärpreventiv behandlingsmodell prövats vid Hjärtenheten, Östersunds sjukhus. Preliminära resultat har presenterats vid Läkarsällskapets riksstämma 2008 (19) och resultat efter en längre tids uppföljning planeras att presenteras under riksstämman 2009. Data talar för att compliance, målvärden och morbiditet väsentligt kan förbättras med denna modell. Detta är observationsdata utan en strikt inklusionsrutin men ger ett övertygande underlag för den nu planerade randomiserade studien med ett strikt protokoll.

**6. Betydelse**

**6.1 Prognosstudien**

Ger möjlighet att direkt mäta skillnad i utfall av tunga händelser (död, stroke, hjärtinfarkt) mellan en uppföljningsrutin baserad på specialiserad sjuksköterska respektive nuvarande gängse uppföljningsrutin i primärvård. Vi vet att de sekundärpreventiva insatserna i dag inte är optimala. Orsakerna är ofullständigt kända men faktorer som bemanningsproblem, många vårdgivare inblandade i beslutskedjan, överbelastning, prioriteringar, logistiska problem med flera spelar in. Ett gynnsamt utfall av sköterskebaserad sekundärprevention med lägre morbiditet och mortalitet skulle motivera ett permanentande och en spridning av denna modell. Betydande positiva effekter både vad gäller hälsa (morbiditet och mortalitet) och ekonomi kan då förutses.

**6.2 Compliance**

Vi har för närvarande mycket bristfälliga kunskaper när det gäller följsamhet till ordinerad medicinering och orsaker till varför ordinationer inte följs. Tillgängliga data talar för att ca 50% av patienterna får problem med att följa given ordination under en 5 årsperiod. Sannolikt kan en stor del av uppkomna problelm lösas och behandlingseffekten bibehållas. Studien kommer att visa om tillgång till respektive monitorering av en specialistsjuksköterska kan öka följsamheten och behandlingseffekten. Populationsaspekten med inklusion av samtliga patienter inom ett definierat upptagningsområde ger studien en unik styrka för validitet och generaliserbarhet.

**6.3 Målvärden**

Guidelines anger rekommenderade målvärden avseende lipider och blodtryck efter hjärtinfarkt respektive stroke. Målvärden sätts med ledning av resultat från stora interventionsstudier. Målvärden för lipider har skärpts och kräver betydande insatser för att nås. Vi har idag inga tillförlitliga data som klarlägger vilken insats som krävs vad gäller patientkontakter, dostitreringar, preparatbyten etc. för att nå aktuella målvärden. I gruppen äldre patienter (>80 år) som helt dominerar när det gäller morbiditet och mortalitet är kunskapen om målvärden och insatser för att nå dessa närmast obefintlig. Studien kommer att ge ett solitt kunskapsunderlag inom området.

**6.4 Kostnads-effektivitetsstudie**

Ger mått på de ekonomiska effekterna av interventionen. Mäts dels omräknat i s.k. QUALY dels direkt mätning av faktisk kostnad alternativt besparing per patient. Hjärtinfarkt och stroke är kostsamt för sjukvården och en reduktion av dessa händelser har potential att ge en betydande besparing som exakt kan kvantifieras i studien.

**6.5 Stressrelaterade faktorer och prognos**

Populationsbaserade, prospektiva data för stressvariablers betydelse för morbiditet och mortalitet efter hjärtinfarkt och stroke saknas.

6.6 Socioekonomi och prognos

Kunskapen om socioekonomiska faktorers betydelse för morbiditet, mortalitet, compliance och målvärden är bristfällig vilket nyligen kommenterats via Socialstyrelsen. En socioekonomisk kartläggning i studien ger underlag för att täppa till denna kunskapslucka.

**7. Tidsplan**

Studien genomförs under perioden 4 januari 2010 t.o.m. 31 december 2014.

**8. Referenser**

1. Socialstyrelsen. Dödsorsaker 2007. Socialstyrelsen 2009.

2. Socialstyrelsen. Hjärtinfarkter 1987-2006. Socialstyrelsen 2008.

3. Årsrapport Riks-Stroke 2007. Riks-Stroke 2008.

4. Socialstyrelsen. Folkhälsorapport 2009. Socialstyrelsen 2009.

5. Stenestrand U, Wallentin L, Lindahl B, Tyden P, Hambraeus K, James S, et al. Årsrapport 2007 - RIKS-HIA, SEPHIA och SCAAR. UCR, Uppsala.; 2008.

6. Wilhelmsson C, Vedin JA, Elmfeldt D, Tibblin G, Wilhelmsen L. Smoking and myocardial infarction. Lancet 1975;2:1157-60.

7. Rockson SG, deGoma EM, Fonarow GC. Reinforcing a continuum of care: in-hospital initiation of long-term secondary prevention following acute coronary syndromes. Cardiovasc Drugs Ther 2007;21(5):375-88.

8. Talelli P, Greenwood RJ. Recurrent stroke: where do we stand with the secondary prevention of noncardioembolic ischaemic strokes? Ther Adv Cardiovasc Dis 2008;2(5):387-405.

9. Bjorck L, Rosengren A, Bennett K, Lappas G, Capewell S. Modelling the decreasing coronary heart disease mortality in Sweden between 1986 and 2002. Eur Heart J 2009;30(9):1046-56.

10. Kotseva K, Wood D, De Backer G, De Bacquer D, Pyorala K, Keil U. Cardiovascular prevention guidelines in daily practice: a comparison of EUROASPIRE I, II, and III surveys in eight European countries. Lancet 2009;373(9667):929-40.

11. Bushnell C, Zimmer L, Schwamm L, Goldstein LB, Clapp-Channing N, Harding T, et al. The Adherence eValuation After Ischemic Stroke Longitudinal (AVAIL) registry: design, rationale, and baseline patient characteristics. Am Heart J 2009;157(3):428-435 e2.

12. Collen A-C, Lagerlöf A, Nieburg I, Carlberg B. Blodtryck efter stroke - hur många når mål? Svenska Läkaresällskapets Riksstämma 2008;Internmedicin:6P.

13. Hanna IR, Wenger NK. Secondary prevention of coronary heart disease in elderly patients. Am Fam Physician 2005;71(12):2289-96.

14. Barter PJ, Caulfield M, Eriksson M, Grundy SM, Kastelein JJ, Komajda M, et al. Effects of torcetrapib in patients at high risk for coronary events. N Engl J Med 2007;357(21):2109-22.

15. Bhatt DL, Fox KA, Hacke W, Berger PB, Black HR, Boden WE, et al. Clopidogrel and aspirin versus aspirin alone for the prevention of atherothrombotic events. N Engl J Med 2006;354(16):1706-17.

16. Thygesen K, Alpert JS, White HD, Jaffe AS, Apple FS, Galvani M, et al. Universal definition of myocardial infarction. Circulation 2007;116(22):2634-53.

17. Osterberg L, Blaschke T. Adherence to medication. N Engl J Med 2005;353(5):487-97.

18. Modica A, Karlsson F, Mooe T. The impact of platelet function or C-reactive protein, on cardiovascular events after an acute myocardial infarction. Thromb J 2009;7:12.

19. Ohlsson-Önerud Å, Mooe T, Modica A, Friberg B, Persson K. KAPRIS - ett Kardiovaskulärt Prevention och Risk Intervenerande System. Läkaresällskapets Riksstämma 2008;Internmedicin:17P.
