# Supplementary material for: Nurse-led secondary preventive follow-up after stroke/TIA and ACS for patients aged 80 years or older: A post-hoc analysis of the randomized controlled NAILED trial
Source: PLoS One. 2025 Nov 7;20(11):e0335930. doi: 10.1371/journal.pone.0335930 (PMC12594373; doi:10.1371/journal.pone.0335930)
Supplement: S2 Table — Categorical values are presented as N (%) and quantitative values as median (interquartile range). TIA, transient ischemic attack; BMI, body mass index; eGFR, estimated glomerular filtration rate; LDL-C, low-density lipoprotein cholesterol; COPD, chronic obstructive pulmonary disease. aLow education level refers to <10 years of education. bIschemic heart disease was defined as a history of myocardial infarction, angina, coronary artery bypass grafting, or percutaneous coronary intervention. (DOCX) [file pone.0335930.s004.docx]

|  | Excluded | Missing |
| --- | --- | --- |
| N | 774 (100) | 0 |
| Women | 454 (58.7) | 0 |
| Age, years | 86.8 (83.9-90.5) | 0 |
| Low education level^a^ | 571 (87.4) | 121 |
| Current/former smoker | 258 (36.2) | 61 |
| **Qualifying event** | |  |
| Unstable angina | 6 (0.8) | 0 |
| Myocardial infarction | 223 (28.8) | 0 |
| Ischemic stroke | 411 (53.1) | 0 |
| Intracerebral hemorrhage | 59 (7.6) | 0 |
| TIA | 72 (9.3) | 0 |
| **Baseline measurements** | |  |
| BMI, kg/m^2^ | 24.1 (21.3-27.1) | 58 |
| eGFR, ml/min | 61.5 (44.3-76.1) | 11 |
| LDL-C, mmol/L | 2.7 (2.1-3.4) | 106 |
| **Medical history** | |  |
| Atrial fibrillation | 306 (39.6) | 1 |
| Ischemic heart disease^b^ | 252 (32.6) | 0 |
| Peripheral artery disease | 27 (3.5) | 0 |
| Diabetes | 157 (20.3) | 0 |
| Congestive heart failure | 139 (18.0) | 0 |
| COPD | 40 (5.2) | 0 |
| Hypertension | 555 (71.7) | 0 |
| Stroke | 170 (22.0) | 0 |
| TIA | 46 (5.9) | 0 |
| **Medications at discharge** | |  |
| Antihypertensive drug | 632 (81.7) | 0 |
| - 1 drug | 196 (25.3) | 0 |
| - 2 drugs | 270 (34.9) | 0 |
| - ≥ 3 drugs | 166 (21.4) | 0 |
| Lipid-lowering drug | 273 (35.3) | 0 |
| Antiplatelet drug | 586 (75.7) | 0 |
| Warfarin | 92 (11.9) | 0 |

**S4 Table. Baseline characteristics of the excluded group.**

Categorical values are presented as N (%) and quantitative values as median (interquartile range). TIA, transient ischemic attack; BMI, body mass index; eGFR, estimated glomerular filtration rate; LDL-C, low-density lipoprotein cholesterol; COPD, chronic obstructive pulmonary disease.

^a^Low education level refers to <10 years of education. ^b^Ischemic heart disease was defined as a history of myocardial infarction, angina, coronary artery bypass grafting, or percutaneous coronary intervention.
