# Supplementary material for: Nurse-led secondary preventive follow-up after stroke/TIA and ACS for patients aged 80 years or older: A post-hoc analysis of the randomized controlled NAILED trial
Source: PLoS One. 2025 Nov 7;20(11):e0335930. doi: 10.1371/journal.pone.0335930 (PMC12594373; doi:10.1371/journal.pone.0335930)
Supplement: S3 Table — HR, hazard ratio; CV, cardiovascular; MI, myocardial infarction. (DOCX) [file pone.0335930.s005.docx]

**S5 Table. Number of patients who reached the primary and secondary endpoints with a maximal follow-up of 1 year.**

|  | Intervention, N (%) | Control, N (%) | Absolute difference (%) | HR (95 % CI) | *P* value |
| --- | --- | --- | --- | --- | --- |
| **Primary endpoint** | | | | | |
| CV death, MI, or stroke | 28 (13.9) | 33 (17.2) | -3.3 | 0.80 (0.48-1.32) | 0.38 |
| **Secondary endpoints** | | | | | |
| CV death | 6 (3.0) | 17 (8.9) | -5.9 | 0.32 (0.13-0.82) | 0.02 |
| MI | 7 (3.5) | 13 (6.8) | -3.3 | 0.50 (0.20-1.24) | 0.14 |
| Stroke | 18 (8.9) | 15 (7.8) | 1.1 | 1.12 (0.57-2.23) | 0.74 |
| All-cause mortality | 14 (6.9) | 24 (12.5) | -5.6 | 0.53 (0.28-1.03) | 0.06 |
| Ischemic stroke | 17 (8.4) | 14 (7.3) | 1.1 | 1.14 (0.56-2.31) | 0.72 |
| Fracture | 15 (7.4) | 4 (2.1) | 5.3 | 3.50 (1.16-10.56) | 0.03 |
| Orthostatic hypotension | 25 (12.4) | 46 (24.0) | -11.6 | 0.48 (0.29-0.77) | < 0.01 |
| Serious bleeding | 8 (4.0) | 4 (2.1) | 1.9 | 1.83 (0.55-6.07) | 0.33 |

HR, hazard ratio; CV, cardiovascular; MI, myocardial infarction.
