# Supplementary material for: Nurse-led secondary preventive follow-up after stroke/TIA and ACS for patients aged 80 years or older: A post-hoc analysis of the randomized controlled NAILED trial
Source: PLoS One. 2025 Nov 7;20(11):e0335930. doi: 10.1371/journal.pone.0335930 (PMC12594373; doi:10.1371/journal.pone.0335930)
Supplement: S4 Table — Values are presented as N (%). (DOCX) [file pone.0335930.s006.docx]

**S6 Table. Number of EQ-5D-3L dimensions and levels during follow-up.**

|  | Intervention | | | Control | | |
| --- | --- | --- | --- | --- | --- | --- |
|  | **1 month** | **3 years** | **5 years** | **1 month** | **3 years** | **5 years** |
| **Mobility** | | | | | | |
| No problems | 86 (48.9) | 45 (40.5) | 24 (39.3) | 91 (54.2) | 58 (51.8) | 22 (46.8) |
| Some problems | 88 (50.0) | 65 (58.6) | 37 (60.7) | 77 (45.8) | 51 (45.5) | 25 (53.2) |
| Extreme problems | 2 (1.1) | 1 (0.9) | - | - | 3 (2.7) | - |
| **Self-care** | | | | | | |
| No problems | 148 (83.6) | 89 (80.2) | 48 (77.4) | 146 (85.9) | 95 (84.8) | 44 (91.7) |
| Some problems | 26 (14.7) | 20 (18.0) | 13 (21.0) | 21 (12.4) | 16 (14.3) | 4 (8.3) |
| Extreme problems | 3 (1.7) | 2 (1.8) | 1 (1.6) | 3 (1.8) | 1 (0.9) | - |
| **Usual activities** | | | | | | |
| No problems | 114 (64.8) | 62 (55.9) | 26 (41.9) | 113 (66.5) | 70 (62.5) | 26 (54.2) |
| Some problems | 55 (31.3) | 43 (38.7) | 35 (56.5) | 47 (27.6) | 38 (33.9) | 22 (45.8) |
| Extreme problems | 7 (4.0) | 6 (5.4) | 1 (1.6) | 10 (5.9) | 4 (3.6) | - |
| **Pain/discomfort** | | | | | | |
| No problems | 73 (41.2) | 50 (45.0) | 25 (40.3) | 69 (40.6) | 50 (44.6) | 26 (54.2) |
| Some problems | 95 (53.7) | 57 (51.4) | 34 (54.8) | 95 (55.9) | 57 (50.9) | 20 (41.7) |
| Extreme problems | 9 (5.1) | 4 (3.6) | 3 (4.8) | 6 (3.5) | 5 (4.5) | 2 (4.2) |
| **Anxiety/depression** | | | | | | |
| No problems | 119 (67.2) | 64 (58.2) | 45 (73.8) | 100 (58.8) | 78 (69.6) | 34 (70.8) |
| Some problems | 55 (31.1) | 43 (39.1) | 14 (23.0) | 69 (40.6) | 32 (28.6) | 14 (29.2) |
| Extreme problems | 3 (1.7) | 3 (2.7) | 2 (3.3) | 1 (0.6) | 2 (1.8) | - |

Values are presented as N (%).
