# Supplementary material for: Nurse-led secondary preventive follow-up after stroke/TIA and ACS for patients aged 80 years or older: A post-hoc analysis of the randomized controlled NAILED trial
Source: PLoS One. 2025 Nov 7;20(11):e0335930. doi: 10.1371/journal.pone.0335930 (PMC12594373; doi:10.1371/journal.pone.0335930)
Supplement: S5 Table — Values are given as N (%). *Any time point before the event. Some events occurred before the first follow-up (1 month). **Any time point after the event. (DOCX) [file pone.0335930.s007.docx]

**S7 Table. Number of participants who provided EQ-5D-3L data.**

|  | Intervention | | Control | |
| --- | --- | --- | --- | --- |
|  | **EQ-5D descriptive system** | **EQ-VAS** | **EQ-5D descriptive system** | **EQ-VAS** |
| Before* a non-fatal primary endpoint | 33 (68.8) | 33 (68.8) | 43 (84.3) | 43 (84.3) |
| After** a non-fatal primary endpoint | 30 (62.5) | 30 (62.5) | 23 (45.1) | 23 (45.1) |
| Before* a fracture | 46 (90.2) | 47 (92.2) | 31 (91.2) | 31 (91.2) |
| After** a fracture | 31 (60.8) | 31 (60.8) | 14 (41.2) | 14 (41.2) |
| ≤ 2 years of follow-up | 187 (92.6) | 187 (92.6) | 173 (90.1) | 174 (90.6) |
| > 2 years of follow-up | 112 (55.4) | 111 (55.0) | 113 (58.9) | 113 (58.9) |

Values are given as N (%). ^*^ Any time point before the event. Some events occurred before the first follow-up (1 month). ^**^ Any time point after the event.
